# Does obesity persist from childhood to adolescence? A 4-year prospective cohort study of chinese students in Hong Kong

**DOI:** 10.1186/s12887-021-02504-7

**Published:** 2021-01-29

**Authors:** Joanna Yuet-ling Tung, Frederick Ka-wing Ho, Keith Tsz-suen Tung, Rosa Sze-man Wong, Wilfred Hing-sang Wong, Bik-chu Chow, Patrick Ip

**Affiliations:** 1 Department of Paediatrics, Hong Kong Children’s Hospital, Kowloon , Hong Kong; 2 Department of Paediatrics and Adolescent Medicine, Queen Mary Hospital, LKS Faculty of Medicine, The University of Hong Kong, Room 123, New Clinical Building, 102 Pokfulam Road, Pok Fu Lam, Hong Kong; 3 Institute of Health and Wellbeing, University of Glasgow, Glasgow, UK; 4 Department of Sports and Physical Education, Hong Kong Baptist University, Kowloon Tong, Hong Kong

**Keywords:** Obesity, Childhood, Obesity consistency, Weight trajectory, Chinese

## Abstract

**Background:**

Little is known about the progression of obesity from childhood to adolescence. This study aimed to longitudinally examine the obesity status in a cohort of children across their childhood and adolescence, and to identify the factors associated with persistent obesity.

**Methods:**

The study used data from School Physical Fitness Award Scheme (SPFAS), a population-based programme in Hong Kong primary and secondary schools. Students were included if they participated in the SPFAS in both 2014 (Primary 1 and 2) and 2018 (Primary 5 and 6). Their anthropometric and physical fitness parameters were analyzed.

**Results:**

A total of 18,863 students were included. The baseline prevalence of obesity was 5.7 %. After 4 years, the prevalence increased to 6.7 %. Among those with obesity at baseline, 35.3 % remained obese after 4 years. The addition of baseline physical fitness level did not improve the prediction for persistent obesity.

**Conclusions:**

One-third of obese students in junior primary school remained to be obese into adolescence. Their baseline physical fitness level did not improve the predictive value for future obesity. Further studies should investigate the prognostic factors that may influence the natural course of childhood obesity.

## Background

Childhood obesity is a significant public health problem worldwide. Obese children have an increased risk of long-term health issues including hypertension, type 2 diabetes, and hyperlipidaemia [1]. Obesity is also associated with adverse consequences including premature mortality and morbidity in adulthood, which has far-reaching direct and indirect social and medical costs [2]. Therefore, it is of utmost importance to prevent childhood obesity and to implement appropriate early interventions. However, most public health programs focused on preventing childhood obesity for the whole population, while much less attention has been paid to planning effective services and interventions for the high-risk group. Given the current childhood obesity epidemic, there have been suggestions to lower the age of referral of obese children, which would potentially allow earlier intervention.

Current guidelines on the referral of obese children in terms of the timing of assessments and/or treatments in primary through to tertiary care are mainly based on expert opinion. The guidelines on obesity management from the UK National Institute for Health and Care Excellence (NICE) recommend that any obese child should be assessed in primary care for community lifestyle modification intervention, whereas those who are extremely/morbidly obese or with any comorbidity should be evaluated in secondary care [3]. The US guidelines recommend children with a BMI greater than or equal to the 85th percentile should be assessed for related morbidities including type 2 diabetes and metabolic syndrome, and should be prescribed ‘intensive, age-appropriate, culturally sensitive, and family-centred lifestyle modifications’ to promote weight loss [4]. The Australian Clinical Practice Guidelines have similar recommendations for specialist referral, but emphasize the role of primary healthcare professionals in monitoring and reviewing the progress after referral [5]. There are no specific recommendations on the cut-off age for referral to specialist care.

In Hong Kong, the Student Health Service (SHS) is the main gatekeeper safeguarding both the physical and psychological health of school-aged children. This is a freely accessible territory-wide health promotion and disease prevention service for all local primary and secondary school children. The SHS provides services that meet the ongoing health needs of enrolled students at various stages of their development, including growth and pubertal assessment, individual health counselling, and health education [6]. Children who are screened to have growth problems, depending on age and severity, will be referred to paediatric units in the Hong Kong Hospital Authority (HA) for secondary care. At present, obese children are referred to HA paediatric units for further management, but the referral guidelines only apply to students ≥10 years of age.

Unquestionably, if the cause of obesity is suspected to be endocrine and syndromic disorders, or if the history suggests comorbidity, then early referral to specialists should be initiated. However, the referral of children with obesity to specialists at a young age might not be absolutely necessary, as obesity-related morbidities are uncommon among asymptomatic obese junior primary school students. Obesity interventions for this age group should mainly focus on diet, physical activity, and behavioural therapy, whereas drug interventions (e.g., metformin, orlistat and sibutramine) are mainly indicated for older obese adolescents [4]. More importantly, although there is an abundance of evidence on adolescent obesity tracking to adulthood, most of these studies mainly focused on children aged 10–12 years at baseline [7, 8]. Little is known about the progression of obesity in junior primary school children aged 6 to 8 years, and the question on whether their obesity will persist into adolescence remains unanswered. Examining the likelihood of persistent obesity into early adolescence among obese junior primary school children would enable us to understand the necessity of early interventions for this age group.

This study aimed to examine the obesity status in middle childhood and early adolescence, and identify factors predicting the persistence of obesity into early adolescence.

## Methods

This study utilized data from the population-based Hong Kong School Physical Fitness Award Scheme (SPFAS) (http://hkchf.hku.hk/). The scheme is jointly organized by the Education Bureau, Physical Fitness Association of Hong Kong, and the Hong Kong Child Health Foundation (HKCHF), and is supported by the Department of Paediatrics and Adolescent Medicine, Queen Mary Hospital, the University of Hong Kong. The SPFAS aims to promote health-related physical fitness among school children and encourage them to participate in regular exercise. Participating students have annual physical fitness and growth assessments at school, mostly in November to January of a school year, and they would be awarded with a certificate if their physical fitness exceeds a certain pre-set level. Currently, more than 400 (~ 50 % across the territory) local primary and secondary schools are involved in this scheme with around 200,000 participating students each year.

### Inclusion and exclusion criteria

Primary school students participating in SPFAS in both 2014 (Primary 1 and 2) and 2018 (Primary 5 and 6) school years were included in the study. A total of 19,242 students with longitudinal identifiers at follow-up were included in the analysis. Those with missing BMI z-score values at baseline (2014) were excluded (n = 379 [2.0 %]) giving an effective study sample size of 18,863.

### Anthropometric parameters

At the start of each academic year, height and weight were measured in students standing barefoot and in light clothing by trained school teachers according to a standardized protocol provided by the panel committee [9]. Body height (to the nearest 0.1 cm) and weight (to the nearest 0.5 kg) were measured using standardized stadiometers and weighing scales calibrated across schools. Body mass index (BMI) was calculated by dividing the weight (in kg) by the square of height (in m). Underweight, overweight, and obesity were defined using the International Obesity Task Force (IOTF) age- and sex-specific cut-off values [10].

### Physical fitness assessment

Annual physical fitness was assessed during physical education classes by trained school teachers according a standardized protocol. The four assessments included (i) handgrip test, (ii) one-minute sit-up test, (iii) sit-and-reach test, and (iv) endurance run (6-minute run for junior primary students and 9-minute run for senior primary students) [9]. These physical fitness assessments have demonstrated good concurrent validity in various health outcomes and are regarded as important health markers. Most of the tests were adopted from the validated EUROFIT test battery [11].

### Statistical analysis

Statistical analysis was performed using statistical software R (R for Windows, version 3.2.4). The data were presented as frequency (percentage) for categorical variables and mean (SD) for continuous variables. For comparisons across age and sex, BMI and fitness test raw values were standardized for age and sex using the lambda-mu-sigma (LMS) method. F-tests and Pearson’s χ^2^ tests were used to assess differences between groups. Poisson regression analysis with robust sandwich estimator was used to examine the association between baseline BMI status (i.e., normal weight vs. overweight vs. obese in 6–8 year olds) and BMI status after 4 years (in 10–12 year olds) adjusted for age, sex, and physical fitness. Receiver operating characteristic analysis was conducted to examine the predictive value of baseline obese status on persisting obesity after 4 years with and without additional physical fitness predictors. Apparent prevalence (% of those screened positive) along with sensitivity, specificity, positive predictive value (PPV), and negative predictive value (NPV) were used to assess the predictive performance. The level of significance was defined as *p* < 0.05.

### Ethics

This study has been approved by Institutional Review Board of the University of Hong Kong and Hong Kong West Cluster, Hospital Authority. Written consent was obtained from schools and parents/guardians before participation in the SPFAS.

## Results

A total of 18,863 students fulfilled the inclusion criteria and were included in the final analyses. Based on the Student Enrolment Statistics published by Hong Kong Education Bureau in 2014/2015, this represented around 16 % of all P.1 and P.2 students (119,941 students) in Hong Kong [12]. Of the 18,863 participating students, 10,513 (55.7 %) were boys and 8,350 (44.3 %) were girls. The prevalence of obesity at baseline (at 5–7 years of age) based on IOTF definition [10] was 5.7 %. After 4 years, the obesity prevalence increased to 6.7 %.

Table 1 summarizes the baseline characteristics of the participants with respect to their weight status at follow-up. In the period between 2014 and 2018, among the 1093 students with obesity at baseline, 396 (35.3 %) remained obese after 4 years; whilst among the 3307 students with either overweight and/or obesity at baseline, 598 (18.1 %) became obese at follow-up 4 years later. A poorer baseline physical fitness level, as indicated by lower overall and individual fitness z-scores, was associated with being obese 4 years later. On the other hand, among the 1272 students who were obese in 2018, 386 (30.3 %) were obese at baseline 4 years earlier.

**Table 1:** Baseline characteristics of participants by BMI status at follow-up after 4 years

Baseline Characteristics	Follow-up Weight Status (After 4 years)				
	**Underweight** **(*n* = 1237)**	**Normal weight** **(*n* = 13,497)**	**Overweight** **(but not obese)** **(*n* = 2857)**	**Obese** **(*n* = 1272)**	***p***
**Sex**^**a c**^					< 0.0001
** Female (*n* = 8350)**	385 (31.1)	6685 (49.5)	971 (34.0)	309 (24.3)	
** Male (*n* = 10,513)**	852 (68.9)	6812 (50.5)	1886 (66.0)	963 (75.7)	
**Baseline Age in years**^**d**^	6.76 (0.62)	6.80 (0.74)	6.78 (0.70)	6.56 (0.64)	< 0.0001
**Baseline BMI z-score**^**d**^	-0.53 (1.46)	0.06 (1.07)	0.75 (1.25)	1.12 (1.51)	< 0.0001
**Baseline Weight Status**					
** Underweight**^**c**^**(*n* = 752)**	188 (15.2)	492 (3.6)	56 (2.0)	16 (1.3)	< 0.0001
** Normal**^**c**^**(*n* = 14,804)**	953 (77.0)	11,411 (84.5)	1782 (62.4)	658 (51.7)	< 0.0001
** Overweight (but not obese)**^**c**^**(*n* = 2214)**	43 (3.5)	1230 (9.1)	729 (25.5)	212 (16.7)	< 0.0001
** Obese**^**c**^**(*n* = 1093)**	53 (4.3)	364 (2.7)	290 (10.2)	386 (30.3)	< 0.0001
** Baseline Fitness Test Overall z-score**^**bd**^	0.14 (0.58)	0.11 (0.64)	0.11 (0.62)	0.05 (0.63)	< 0.0001
**Baseline Fitness Test z-score**					
** Grip strength**^**d**^	0.05 (1.09)	0.15 (1.00)	0.41 (1.11)	0.25 (1.12)	< 0.0001
** Sit-up**^**d**^	0.05 (1.12)	-0.03 (1.12)	-0.20 (1.21)	-0.32 (1.30)	< 0.0001
** Sit and reach**^**d**^	-0.11 (1.02)	-0.10 (1.09)	-0.37 (1.42)	-0.46 (1.54)	< 0.0001
** Endurance run**^**d**^	0.55 (0.84)	0.36 (0.99)	0.17 (0.90)	0.18 (0.84)	< 0.0001

^a^Sample sizes for females and males were 8,460 and 10,660, respectively

^b^Average of all four fitness tests

^c^ n (%), comparisons were made using Pearson’s χ2 tests

^d^ Mean (SD), Comparisons were made using F-tests

Obese status at baseline was found to be associated with obese status after 4 years at follow-up (prevalence ratio, PR = 6.48, *p* < 0.0001) after adjusting for age and sex. The second strongest factors associated with obese status at follow-up was overweight status at baseline (PR = 3.91, *p* < 0.0001). Baseline BMI z-score (PR = 1.73) was a stronger factor in association with future weight status than any of the physical fitness indicators, with PRs ranging from 0.76 to 1.02 (Table 2). However, further interaction analysis revealed that age was a significant moderator for the association. For each increment in age (year), the prevalence ratio (PR) of baseline obesity increased by 40 %. This was shown by the predictive value of obese status increasing from 6 to 8 years of age (ranging from 0.180 to 0.304 in females and 0.432 to 0.729 in males), suggesting older age at baseline was more associated with obese status at follow-up. For 8-year-olds, 72.9 % of obese boys were persistently obese at follow-up compared with 30.4 % of obese girls with a PR of 2.40 (Table 3).

**Table 2:** Poisson regression analysis estimating relative risks of obese status at follow-up by baseline characteristics

Baseline Characteristics	PR (95 % CI)	*p*
BMI z-score	1.73 (1.66, 1.80)	< 0.0001
Underweight	0.38 (0.23, 0.62)	< 0.0001
Overweight	3.91 (3.49, 4.38)	< 0.0001
Obese	6.48 (5.69, 7.39)	< 0.0001
Overall fitness	0.81 (0.74, 0.90)	< 0.0001
Grip strength	1.02 (0.96, 1.08)	0.60
Sit-up	0.92 (0.87, 0.97)	0.004
Sit and reach	0.99 (0.94, 1.05)	0.79
Endurance run	0.76 (0.71, 0.81)	< 0.0001

*PR* prevalence ratio, a relative risk measure as the ratio of obesity prevalence at follow-up

**Table 3:** Predicted prevalence (%) of obese status at follow-up after 4 years by baseline non-obese and obese status

	Non-obese at baseline			Obese at baseline		
	**Obesity Prevalence after 4 years**	**95 % ** **Lower CI**	**95 % ** **Upper CI**	**Obesity Prevalence after 4 years**	**95 % ** **Lower CI**	**95 % ** **Upper CI**
**Female**						
6 years old at baseline	3.0	2.6	3.3	18.0	15.3	21.2
7 years old at baseline	1.6	1.4	1.9	23.4	19.2	28.4
8 years old at baseline	0.9	0.7	1.2	30.4	22.1	41.9
**Male**						
6 years old at baseline	7.1	6.6	7.7	43.2	38.1	49.0
7 years old at baseline	3.9	3.4	4.5	56.1	47.4	66.5
8 years old at baseline	2.1	1.7	2.7	72.9	53.5	99.2

Predicted using the Poisson model shown in Table 2

Adding the baseline physical fitness level reduced the predicted prevalence, improved the specificity, and reduced the sensitivity (Table 4). There were no substantial changes in PPV and NPV after adding baseline physical fitness predictors.

**Table 4:** Receiver operating characteristics analysis examining the predictive performance of baseline obese status and additional fitness parameters on follow-up obese status

Models	Predicted prevalence	Sensitivity	Specificity	PPV	NPV
**1: Obese at Baseline**	0.05 (0.04–0.05)	0.25 (0.23–0.28)	0.97 (0.96–0.97)	0.33 (0.30–0.36)	0.95 (0.95–0.95)
**2a: + Overall Fitness**	0.02 (0.02–0.02)	0.10 (0.08–0.11)	0.99 (0.98–0.99)	0.31 (0.26–0.36)	0.94 (0.94–0.95)
**2b: + Endurance run**	0.02 (0.02–0.02)	0.13 (0.11–0.15)	0.98 (0.98–0.99)	0.35 (0.31–0.40)	0.94 (0.94–0.95)
**2c: + Sit-and-reach**	0.03 (0.03–0.03)	0.15 (0.13–0.17)	0.98 (0.98–0.98)	0.32 (0.28–0.36)	0.95 (0.94–0.95)
**2d: + Sit-up**	0.02 (0.02–0.03)	0.13 (0.11–0.15)	0.98 (0.98–0.99)	0.35 (0.30–0.39)	0.94 (0.94–0.95)
**2e: + hand grip**	0.02 (0.01–0.02)	0.05 (0.03–0.06)	0.99 (0.98–0.99)	0.18 (0.14–0.23)	0.94 (0.94–0.94)

*PPV* positive predictive value; *NPV* negative predictive value

Note that Model 1 has only one predictor, Obese at Baseline, whereas Models 2a to 2e also include an additional fitness predictor

## Discussion

To the best of our knowledge, only limited studies examined the persistence of obesity from middle childhood to early adolescence. In this study, we found that obese status at baseline was the strongest predictor for obese status at the 4-year follow-up after adjusting for age and sex. This finding highlighted the potential of delivering obesity interventions to students at an earlier age such as those in junior primary schools. However, when we examined the persistence of obesity in these young children, only around 35 % remained obese after 4 years. In other words, after 4 years, around 65 % of the obese junior primary students in this study became non-obese in the absence of specific obesity interventions except participation in the SPFAS program. Nevertheless, this is not trivial at a population level. Notably, age was found to be a significant moderator for this association. The predictive value of obese status at baseline increased from 6 to 8 years, suggesting that older age at baseline was more predictive of subsequent obese status. Furthermore, we found that the baseline physical fitness level did not change the stable progression of obesity in the 4-year study period.

Although numerous studies have examined the persistence of obesity from childhood to early adulthood, most of these studies focused on early adolescents. For example, in a 10-year follow-up study of 2,124 adults, Freedman et al. demonstrated a moderate correlation (r = 0.60) of body mass index (BMI) in childhood and adulthood. However, the median age of this cohort at baseline was approximately 11 years [7]. On the other hand, the National Longitudinal Survey of Youth (NLSY79) in the US found that around 9 % were chronically obese across childhood into adolescence, and 11 % became non-obese in their early adolescent years [13]. This study examined BMI developmental trajectories in subjects born across two decades between 1986 and 2008. Substantial environmental and lifestyle changes could have occurred in recent years, making it difficult to apply the findings in a contemporary world with obesogenic environments. Although there are more longitudinal studies on group-level BMI trajectories from childhood to adolescence, individual-level evidence remains scant. These studies, in general, identified four to five obesity trajectories and around 6–13 % of the children would remain obese across childhood into early adolescence [14, 15]. However, the use of these group-based BMI developmental trajectories may have issues related to ecological fallacy, given that they can only assess the average pattern of change over time and categorize children into different pre-defined sub-groups without considering potential individual-level variations.

Similar to our study, a recent population-based longitudinal study by a German group examined the risk of sustained obesity between early childhood and adolescence and found that the majority of children (approximately 50 %) who were obese at 5 years of age remained obese into adolescence [16]. Their findings are slightly higher than our findings that approximately 35 % of obese junior primary students remained obese into early adolescence. Previous studies reported that ecologically relevant factors, including parental education, overweight parents, physical activity, and screen time, were associated with ‘persistently obese’ trajectories [14, 17, 18]. Other potential reasons include ethnicities [19] or living environment which can also influence growth patterns. Therefore, in order to ascertain the critical periods of sustained childhood obesity, a more holistic approach should be adopted to consider the effects of ecological factors at different levels, including individual (e.g., ethnicity), interpersonal, community, organizational, public policy, and the enabling environment [20, 21], as well as judicious use of overseas data. Such approach would enable the formulation of more effective public health programs or interventions for childhood obesity.

Socioeconomic positions, parental factors and other lifestyle habits are well known to be associated with obesity in children [22, 23]. Unfortunately, all these data are not routinely collected in the SPFAS, and hence could not be analysed in this study. In fact, such information was not collected in the routine health checks for students as well. Currently, guidelines on referral for further obesity intervention from SHS are mainly based on weight status, rather than integrating all the aforementioned factors. This demonstrates the gap in integrating the scientific evidence with real-world practice. Our study had shown that, the use of weight status and physical fitness parameters alone, would be inadequate to further categorise the highest risk group for further interventions. This, hopefully, could alert the policy makers that a more holistic approach integrating all these factors would be needed in the planning and development of childhood obesity programmes for at-risk groups.

Our cohort was drawn from SPFAS, a physical fitness programme that aims to promote physical activity among students. We observed a relatively low rate of sustained obesity from middle childhood to adolescence, this could possibility be partly related to the effectiveness of this programme. Further studies would be useful to determine the contribution of this fitness programme towards the progression of obesity in this group of children, as well as to determine which intervention components are the most effective. Various studies have reported effects of gender on BMI trajectory in school children, but these findings have been inconsistent. Some studies showed no gender effects [14], whereas some studies demonstrated that boys were more likely to be more chronically obese [13–24]. Interestingly, we observed significant gender differences in the persistence of obesity from childhood to early adolescent years, with a higher rate of persistent obesity observed in boys. A similar gender-specific BMI developmental trajectory was also found in a Taiwanese cohort [18]. It has been postulated that girls may pay more attention to their body image and thus make greater efforts to control their weight [25]. On the other hand, it might also imply that, BMI may not be the most accurate proxy for adiposity among adolescence, with girls developing more adiposity while boys developing more muscle mass at this stage.

The strengths of this study include a large sample size (~ 16 % of the population) with a longitudinal follow-up of 4 years spanning the period between childhood and early adolescence. We utilized longitudinal data from SPFAS to assess the persistence of obesity among junior primary school students. This study sample is reasonably representative of all children in Hong Kong, as SPFAS has been adopted by more than 50 % of all local primary schools and covers a range of socio-economic positions (SEP) across Hong Kong. In addition to anthropometric measures, data on their physical fitness levels were also available. However, the findings should be interpreted with the following caveats. First, data on birth weight and early infant growth patterns were not available. Systematic reviews showed that high and low birth weight, as well as early rapid weight gain, were positively associated with the development of overweight and obesity in later life [26, 27]. Second, data on their pubertal status, socioeconomic positions, parental weight, dietary intake, and physical activity were not available. Third, students were not evaluated for obesity-related disordered such this endocrinopathies or syndromic obesity. However, all students were recruited from mainstream, normal schools and hence the chance of having syndromic obesity, which typically is associated with developmental delay or visual problem, would be relatively low. Lastly, the findings were limited by missing data, although the missing data rate of 2.0 % was relatively low.

## Conclusions

This study found that 35 % of junior primary school students remained obese in early adolescence with a higher risk of persistent obesity observed in boys. This is not trivial when considered at a population level. The baseline BMI z-score was the strongest predictor for persistent obesity, while the baseline physical fitness level did not increase the model predictive value. Future studies should explore other prognostic factors that may influence the persistence of obesity in children. Our findings can inform the planning and development of childhood obesity programmes for at-risk groups. In particular, children who have a higher risk for persistent obesity such as obese young boys should be referred for early interventions.

## Data Availability

The datasets used and/or analysed during the current study are available from the corresponding author on reasonable request.

## Abbreviations

BMI: Body mass index
CI: Confidence interval
HA: Hospital Authority
IOTF: International Obesity Task Force
NPV: Negative predictive value
PPV: Positive predictive value
PR: Prevalence ratio
SD: Standard deviation
SEP: Socio-economic position
SHS: Student Health Service
SPFAS: School Physical Fitness Award Scheme
